# Translating GWAS Findings to Inform Drug Repositioning Strategies for COVID-19 Treatment

**DOI:** 10.21203/rs.3.rs-3443080/v1

**Published:** 2023-10-19

**Authors:** Ming-Ju Tsai, Sohyun Jeong, Fangtang Yu, Ting-Fu Chen, Peng-Hsuan Li, Hsueh-Fen Juan, Jia-Hsin Huang, Yi-Hsiang Hsu

**Affiliations:** Hebrew SeniorLife; Hebrew SeniorLife; Hebrew SeniorLife; Taiwan AI Labs; Taiwan AI Labs; National Taiwan University; Taiwan AI Labs; Broad Institute

**Keywords:** SARS-COV-2, GWAS, post-GWAS, Gene prioritization, Drug repurposing, molecular docking, COVID-19

## Abstract

We developed a computational framework that integrates Genome-Wide Association Studies (GWAS) and post-GWAS analyses, designed to facilitate drug repurposing for COVID-19 treatment. The comprehensive approach combines transcriptomic-wide associations, polygenic priority scoring, 3D genomics, viral-host protein-protein interactions, and small-molecule docking. Through GWAS, we identified nine druggable host genes associated with COVID-19 severity and SARS-CoV-2 infection, all of which show differential expression in COVID-19 patients. These genes include IFNAR1, IFNAR2, TYK2, IL10RB, CXCR6, CCR9, and OAS1. We performed an extensive molecular docking analysis of these targets using 553 small molecules derived from five therapeutically enriched categories, namely antibacterials, antivirals, antineoplastics, immunosuppressants, and anti-inflammatories. This analysis, which comprised over 20,000 individual docking analyses, enabled the identification of several promising drug candidates. All results are available via the DockCoV2 database (https://dockcov2.org/drugs/). The computational framework ultimately identified nine potential drug candidates: Peginterferon alfa-2b, Interferon alfa-2b, Interferon beta-1b, Ruxolitinib, Dactinomycin, Rolitetracycline, Irinotecan, Vinblastine, and Oritavancin. While its current focus is on COVID-19, our proposed computational framework can be applied more broadly to assist in drug repurposing efforts for a variety of diseases. Overall, this study underscores the potential of human genetic studies and the utility of a computational framework for drug repurposing in the context of COVID-19 treatment, providing a valuable resource for researchers in this field.

## Introduction

The COVID-19 pandemic has presented significant challenges in finding appropriate treatments. Over 620 drug development programs have been reported, with numerous antivirals, cell and gene therapies, immunomodulators, and neutralizing antibodies being investigated ^1^. Drug repurposing is a cost-effective and expedited approach to finding effective therapeutic agents^2^,^3^. Approved drugs, such as interferon, chloroquine, remdesivir, lopinavir, ritonavir, and angiotensin receptor blockers, have been studied for their potential to combat COVID-19. Remdesivir, initially evaluated in clinical trials for the Ebola outbreak, has been approved to treat COVID-19 patients. Recent studies have shown that Remdesivir reduced respiratory infection and shortened recovery time in hospitalized COVID-19 adults^4^. The FDA has approved Remdesivir for COVID-19 treatment in hospitalized patients with positive SARS-CoV-2 viral testing results. Another repurposed drug is Olumiant, a Janus Kinase inhibitor used to treat severe alopecia areata, which has been approved to treat severe COVID-19 in hospitalized adults. Combining Olumiant and Remdesivir has further shortened recovery time and improved clinical status ^5^.

Human genetics studies have long been used to identify the pathophysiology of diseases and novel therapeutic targets for various diseases, such as Type 2 diabetes, rheumatoid arthritis, ankylosing spondylitis, psoriasis, osteoporosis, schizophrenia, and dyslipidaemia^6^. With the COVID-19 pandemic, researchers are conducting genome-wide association studies (GWAS) to identify host genetic susceptibility to the virus and its causative agent, SARS-CoV-2. Recently, GWAS loci on chromosome 3p21.31 and 9q34.2 have been associated with respiratory failure in 1,980 severe COVID-19 patients compared to 2,381 control subjects from Italy and Spain ^7^. The COVID-19 Host Genetics Initiative (COVID-19 HGI) has conducted meta-analyses of 46 GWAS from 19 countries with different genetic ancestries, providing insights into the host genetic effects of COVID-19 ^8^. Gene prioritization in GWAS is an important therapeutic strategy to identify the latest and most effective treatment targets^9^. Understanding virus-host gene interactions is essential to determine how viruses infect host and replicate within them, and how the host immune system responds to pathogens. Gordon D et al. generated a SARS-CoV-2 virus-host interactome, containing 332 high-confidence protein-protein interactions between SARS-CoV-2 virus proteins and human proteins using affinity purification mass spectrometry. This interactome serves as a valuable reference for prioritizing host genes as potential targets for treating COVID19^10^.

We present here a novel computational framework designed to facilitate drug repurposing for the treatment of COVID-19. This framework leverages a combination of transcriptomic-wide associations, polygenic priority score, 3D genomics, viral-host protein-protein interactions, and small-molecule docking techniques to identify potential drug candidates. Using a GWAS approach, we identified nine druggable host genes that are associated with COVID-19 severity and SARS-CoV-2 infection, and are differentially expressed in COVID-19 patients. Subsequent molecular docking analysis of 553 small molecules from five therapeutic categories, including antibacterial, antiviral, antineoplastic, immunosuppressant, and anti-inflammatory drugs, enabled us to identify several promising drug candidates, such as Peginterferon alfa-2b, Interferon alfa-2b, Interferon beta-1b, Ruxolitinib, Dactinomycin, Rolitetracycline, Irinotecan, Vinblastine, and Oritavancin.

Importantly, our proposed framework offers a versatile approach that can be applied beyond COVID-19, to aid in drug repurposing efforts for other diseases. Our study emphasizes the potential of human genetics studies and bioinformatics analysis in prioritizing gene targets, which can be further evaluated through molecular docking analyses with known and approved drug molecules. By providing a comprehensive approach to prioritize gene targets from GWAS studies, our framework enables researchers to identify underlying causal genes within each GWAS locus and to further prioritize them based on expression levels, tissue-specificity, and enrichment analyses.

## Results

### Identification of target genes associated with COVID-19 relevant phenotypes

To identify target genes associated with COVID-19 relevant phenotypes, we employed five different methods, including Transcriptome-wide associations study (TWAS)^11^, polygenic priority score (PoPS)^12^, Activity-by-contact (ABC) model^13^, Capture Hi-C Omnibus Gene Score (COGS)^14^, and nearest genes with protein-protein interactions (PPIs). Using all GWAS summary statistics from COVID-19 HGI, we prioritized 41 genes. Of these, 25 were found in 11 GWAS significant loci (Table 1), and 16 were found in 16 suggested loci (Supplementary Table 1). Analysis of the prioritized genes showed that 17 genes were associated with critical illness at eight loci, including 3p21.31, 6p21.33, 12q23.2, 19p13.3, 7p11.2, 21q22.11, 19p13.2, and 12q24.13 with four critical illness-specific genes (*XCR1, TYK2, FDX2*, and *ICAM4*). Additionally, we identified 16 genes associated with hospitalization at eight loci, including 3p21.31, 6p21.33, 12q23.2, 19p13.3, 7p11.2, 21q22.11, 6p21.1, and 12q24.13 with three hospitalization-specific genes (*FOXP4, OAS1*, and *OAS2*). We also found 12 genes associated with infection at six loci, including four infection-specific genes (*TCF19, ABO*, *OBP2B*, and *TLE1*). In particular, the PPI analysis of *TLE1* revealed it interacts with the SARS-CoV2 nsp13 protein.

### Identification of repurposing drug categories using Gene-set enrichment analysis

In order to gain further insight into the biological functions of the 41 prioritized genes, we performed gene set enrichment analysis using four gene sets libraries containing these genes. The enriched gene sets were identified using a combination of the ranked combined score and adjusted p-value cutoff of 0.05. We identified six gene sets from the WikiPathways database (Fig. 2.a), five from the Elsevier database (Fig. 2.b), five from the Gene Ontology (GO) molecular function category (Fig. 2.c), and 12 from the GO cellular component category (Fig. 2.d). The details of enriched gene sets are shown in Supplementary Tables 3 to 6.

After conducting a manual review of the top enriched biological mechanisms and pathways in each library, we identified five drug categories: antibacterial, antiviral, antineoplastic, immunosuppressive, and anti-inflammatory. To further investigate the potential for molecular docking of these drugs with the prioritized genes, we collected molecules from ChEMBL database in these five categories.

### Identification and functional characterization of druggable targets for COVID-19

A total of 22 druggable targets were identified by DGIdb, including IFNAR1, IFNAR2, TYK2, SLC6A20, CCR9, CXCR6, XRC1, IGF1, IL10RB, OAS1, OAS2, OAS3, GRIK4, FMO5, ST3GAL1, GAS7, TRIM33, DPP9, MAT2B, ICAM44, OBP2B, TRHR. Since IFNAR1, IFNAR2, and TYK2 already have approved drugs, the functional characterized analysis will focus on the remaining 19 druggable targets. Through COVID-19-relevant transcriptome and enrichment gene sets, seven druggable targets with COVID-19-relevant functions were identified, including CCR9, CXCR6, XCR1, IL10RB, OAS1, OAS2, and OAS3. The details of functional annotation results are shown in Supplementary Table 7.

### Molecular docking with a searchable docking browser

The study collected drug ligands from ChEMBL in five categories: antibacterials (n = 221), antivirals (n = 67), antineoplastics (n = 172), immunosuppressants (n = 22), and anti-inflammatory (n = 71). We conducted comprehensive molecular docking for all 41 prioritized genes. More than 20,000 molecular docking analyses were conducted in this study. All docking results are available in the DockCoV2 database (https://dockcov2.org/drugs/). DockCoV2 also provides literature mining related to COVID-19 for interesting genes or compounds. The detail of DockCoV2 implementation is in the supplemental information.

### Discovery of repurposable drugs from prioritized drug-gene interactions for COVID-19

The approval drug-gene interactions are shown in Table 2. Peginterferon alfa-2b (DrugBank ID: DB00022), Interferon alfa-2b (DrugBank ID: DB00105), and Interferon beta-1b (DrugBank ID: DB00068) are all approved drugs that target IFNAR1 and IFNAR2. Peginterferon alfa-2b is an antiviral and immunoregulatory drug that stimulates the innate antiviral response in treating hepatitis B, C, and some cancers. Interferon alfa-2b is recombinant human interferon used to treat hepatitis B and C infection, genital warts, hairy cell leukemia, follicular lymphoma, malignant melanoma, and AIDs-related Kaposi’s sarcoma. Interferon beta-1b is another form of recombinant human interferon used to slow the progression of relapsing multiple sclerosis and reduce the frequency of clinical symptoms. Ruxolitinib, an approved drug that targets TYK2, is a kinase inhibitor used to treat patients with myelofibrosis and polycythemia vera who have not responded to or are unable to tolerate the use of hydroxyurea, and to treat graft-versus-host disease when steroid treatments have failed. The prioritized drug-gene interactions are shown in Table 3 based on the COVID-19 GWAS analysis, strong binding from molecular docking analysis (Binding affinity < −10 kcal/mol), and relevant literature: Dactinomycin (DB00970), Rolitetracycline (DB01301), Irinotecan (DB00762), Vinblastine (DB00570), and Oritavancin (DB04911).

## Methods

### COVID-19 GWAS and genomic risk loci

The COVID-19 Host Genetics Initiative (COVID-19 HGI) represents a consortium of over 1000 scientists from over 54 countries working collaboratively to share data, recruit patients, and disseminate novel findings^8^. This study used COVID-19 HGI GWAS meta-analyses round 4 (Sep 30, 2020) to perform downstream analyses. There are six cohorts, including A2) very severe respiratory confirmed COVID (n = 4,933) verse population (n = 1,398,672); B1) hospitalized COVID (n = 2,430) vs. not hospitalized COVID (n = 8,478); B2) hospitalized COVID (n = 8,638) verse population (n = 1,736,547); C1) COVID verse lab/self-reported negative; C2) COVID (n = 30,937) verse population (n = 1,471,815); D1) Predicted COVID from self-reported symptoms (n = 3,024) verse predicted or self-reported (n = 35,728). The COVID-19 HGI has identified six phenotypes that can be used to group individuals into three distinct disease statuses: critical illness, hospitalization, and reported infection^8^. A2 with very severe respiratory confirmed COVID was classified as critical illness, B1 and B2 were classified as hospitalization, and C1 and C2 were classified as reported infection. In order to further understand the genetic basis of these disease statuses, we identified significant independent SNPs with a genome-wide significant *P*-value (< 5×10^−8^) and independent from each other at r^2^ < 0.6. Furthermore, if the linkage disequilibrium (LD) blocks of significant independent SNPs are closely located to each other (< 250 kb based on the most right and left SNPs from each LD block), they were merged into one genomic locus.

### A computational framework for prioritizing the drug-gene interactions

**Figure 1:**
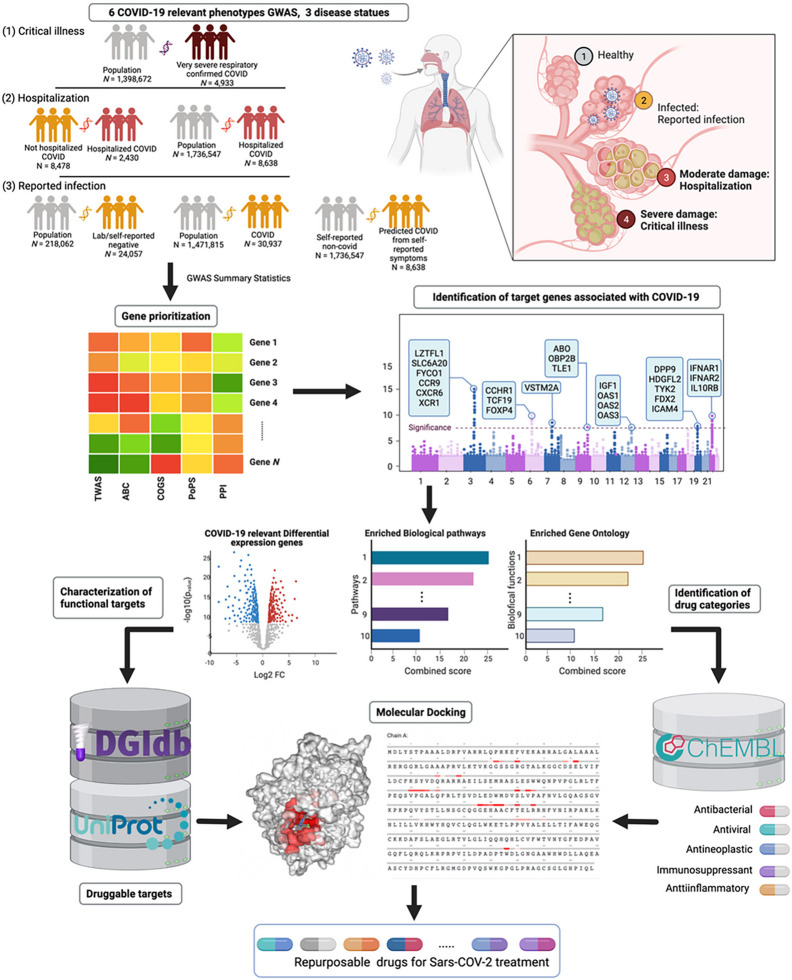
The computational framework for prioritizing drug-gene interactions from GWAS summary statistics.

**Figure 2:**
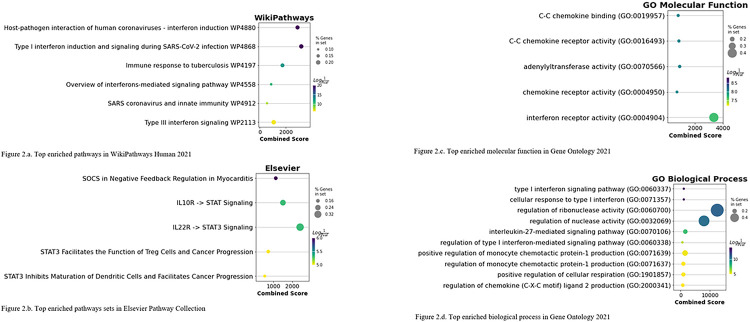
The results of enrichment analysis in four different gene-set libraries.

We propose a computational framework for prioritizing drug-gene interactions relevant to COVID-19. This framework consists of four main components: gene prioritization, identification of drug categories, target functional characterization, and comprehensive docking analysis and literature review (Fig. 1). Gene prioritization uses five different methods to prioritize target genes relevant to COVID-19 phenotypes and all details were in supplementary. Drug categories are identified using gene-set enrichment analyses. Target functional characterization is performed by analyzing differential gene expression in cells and tissues due to SARS-CoV-2 RNA-seq profiles, genes with significant TWAS results in the lung or whole blood tissues, and genes involved in the top gene-set enrichment results. Finally, comprehensive docking analysis and literature review are used to identify highly druggable drug-gene interactions for COVID-19 drug repurposing.

### Gene prioritization from COVID-19 GWAS loci

In our rigorous gene prioritization approach, multiple facets of gene prioritization are considered. We employed five state-of-the-art methods to discern target genes within each GWAS locus: 1) transcriptome-wide association study (TWAS); 2) Polygenic Priority Score (PoPS); 3) 3D chromosomal topology, Activity by Contact (ABC) Model of Enhancer-Gene; 4) Capture Hi-C Omnibus Gene Score (COGS); 5) Nearest gene with Protein-Protein Interactions (PPIs) within each GWAS significant and suggestive locus. In-depth explanations for each method are provided in the supplementary materials.

### Gene-Set Enrichment Analysis

We performed a gene-set enrichment analysis to identify enriched pathways and functions to explore potential drug categories from gene prioritization results. We used GSEApy^15^ and Enrichr^16,17^ to analyze gene-set enrichment. Each gene set was evaluated by combining the p-value computed using the Fisher exact test with the z-score of the deviation from the expected rank by multiplying these two numbers as follows:

c=log(p)·z

This study used four gene-set libraries, including 1) WikiPathways Human 2021^18^, 2) Elsevier Pathway Collection by Enrichr^17^, 3) Gene Ontology Molecular Function 2021^19^, and 4) Gene Ontology Biological Process^19^.

### Functional Characterization of druggable targets relevant to COVID-19

The druggability of the target is critical for drug repurposing. The Drug Gene Interaction Database (DGIdb)^20^ organizes genes with known drug interactions obtained from literature or publicly available databases, as well as genes that may be druggable according to categories such as kinases, druggable genome, cell surface, and transcription factor. We used the “druggable genome” category to identify potentially druggable targets among target genes from gene prioritization results. Furthermore, characterizing the functional druggable target genes and assessing whether they are relevant to COVID-19 pathways or biological mechanisms is crucial. We utilized three functional resources, including 1) differentially expressed genes (DEGs) from COVID-19-related RNA-seq profiles (Supplementary Table 7) or TWAS results from lung and whole blood tissues; 2) the enriched pathways from WikiPathways Human and Elsevier Pathway; 3) enriched gene sets from Gene Ontology. The functional targets must be present in at least two functional resources.

### Comprehensive Molecular Docking

We performed molecular docking to prioritize drug-gene interactions using potential drugs identified through enrichment analysis and functional druggable genes identified through GWAS. A structure-data file (SDF) containing the three-dimensional structure of each drug was obtained from the ChEMBL^21^ database. The Protein sequences of the functional druggable genes were collected through the UniProt^22^ database and used them as templates to build approximate structure in SDF format using Swiss-model^23^.

This study used the CB-dock framework^24^ to predict the binding sites for a given protein using a novel curvature-based cavity detection approach, and calculated the centers and sizes of these binding sites. Within the CB-dock framework, we used QuickVina2 to perform a large-scale molecular docking analysis^25^. QuickVina2 enhances speed through the use of heuristics that prevents unnecessary local searches. Since QuickVina2 only accepts inputs in the Protein Data Bank, Partial Charge (Q), and Atom Type (T) (PDBQT) format, we used OpenBabel^26^ to convert SDF files to PDBQT format. The docking results were further processed to build a protein heatmap corresponding to the affinity score with the ligand docking positions described previously^27^. Next, we updated all docking results in the DockCoV2 database^27^.

## Discussion

In this study, we developed a computational framework to translate GWAS findings into drug repositioning for the treatment of COVID-19. Our approach began by utilizing five state-of-the-art methods to identify 41 prioritized genes associated with COVID-19 infection and/or severity. We then further refined this list by identifying 10 druggable gene targets among the 41 prioritized genes, based on their functions in DGIdb, COVID-19 DEGs, and TWAS results from lung and whole blood tissues, as well as enrichment gene sets. According to the enriched gene sets, we collected 553 drugs/ligands from the five treatment categories, including antibacterial, antiviral, antineoplastic, immunosuppressive, and anti-inflammatory. Our computational framework then identified night drugs/ligands based on their mechanism of action or molecular docking, followed by a review of their known toxicities in the literature and drug databases. The night potential drugs for COVID-19 treatment are Peginterferon alfa-2b, Interferon alfa-2b, Interferon beta-1b, Ruxolitinib, Dactinomycin, Rolitetracycline, Irinotecan, Vinblastine, and Oritavancin.

Out of the night drugs identified in our computational framework, four drugs with clear drug-gene interactions: Peginterferon alfa-2b (DB00022), Interferon alfa-2b (DB00105), Interferon beta-1b (DB00068), and Ruxolitinib (DB08877). Interferon alfa-2b, peginterferon alfa-2b, and interferon beta-1b are associated with Type I interferon (IFN) responses, which are the primary defense against viral infection. Gene-Set enrichment results showed that interferon receptor activity from Gene Ontology (GO:0004904, Odds Ratio:332.58) and Type 1 interferon induction and signaling during SARS-CoV-2 infection (WP4868, Odds Ratio:132.89) were highly enriched. A number of viruses, including SARS-CoV-2, have evolved mechanisms that enable them to evade the antiviral effects of Type I interferon (IFN-I)^28^. Numerous experiments have been conducted in both vitro and in vivo to study the efficacy of IFN-I treatment against MERS-CoV and SARS-CoV^29^. The Janus family kinases (JAK) and tyrosine kinase 2 (TYK2) are associated with the membrane-proximal part of the cytoplasmic domains of IFNAR1 and IFNAR2, respectively^28^. *TYK2* is a prioritized target gene in this study and has an approved immunosuppressant named Ruxolitinib. TYK2 and IL10RB are in Type III interferon signaling, and IL10RB is the top key regulator of COVID-19 host susceptibility^30^.

Five highly druggable drug-gene interactions for COVID-19 drug repurposing were identified from functional analyses, relevant literatures, and docking analysis (Table 3): Dactinomycin, Rolitetracycline, Irinotecan, Vinblastine, and Oritavancin. Datinomycin binds IL10RB strongly (binding affinity: −13.5 kcal/mol) and has been widely used as an inhibitor of viral cellular transcription in infected cells^31^. The combination of dactinomycin and sirolimus exhibited synergistic effects against the host proteins of SARS-CoV-2.^32^. Oritavancin strongly binds with OAS1 (binding affinity: −16.6 kcal/mol) and has also been suggested as a potential COVID-19 treatment option that inhibits cathepsin L and cathepsin B in host cells (late endosomal pathway)^33^. Irinotecan is a topoisomerase I inhibitor that binds to CCR9 protein (binding affinity: −17.1 kcal/mol) and has also been suggested as a potential candidate therapy to counter cytokine storms in critically ill COVID-19 patients^34^. XCR1 has a strong binding affinity with vinblastine (binding affinity: −16.3 kcal/mol), and vinblastine showed robust anti–SARS-CoV-2 response in human VeroE6 cells^35^.

The challenge in post-GWAS is to identify the causal variants and their target genes within the GWAS locus. To address this challenge, many advanced approaches have been developed, such as fine-mapping, functional annotation, and gene prioritization^36,37^. In this study, we applied state-of-the-art gene prioritization methods to prioritize target genes from COVID-19 GWAS. These approaches have different perspectives on prioritizing target genes, including gene-based methods that use transcriptome gene expression coupled with genetic variants from GWAS, enhancer-promoter identification based on three-dimensional chromatin interactions data (e.g., Hi-C, Capture Hi-C), and open chromatin information, and similarity-based methods that incorporate data from publicly available RNA-seq datasets, curated biological pathways, and protein-protein interaction data.

Further cellular and animal experiments are needed to validate our findings. The percentage of drug mechanisms directly supported by human genetic studies increases throughout the drug development pipeline. Therefore, selecting genetically supported drug targets could improve the success rate of drug development and reduce the time and costs of developing new drugs^38^. In summary, this study presents a bioinformatics framework for drug repurposing to translate GWAS findings and provides druggable candidates in immune reactions and host-viral interaction pathways. In addition to COVID-19, this computational framework can be applied to other GWASs for drug repurposing to speed up the discovery of potential therapeutics.

## Figures and Tables

**Table 1:** 25 Prioritized genes located in GWAS significant loci

Cytoband	Lead SNP	Target gene	Types of gene prioritization methods	Phenotypes of COVID-19
			TWAS	POPS	ABC	COGS	PPI	Critical illness	Hospitalized	Reported infection
3p21.31	rs35081325	LZTFL1	V	V	V		V	V	V	V
		SLC6A20	V	V	V	V		V	V	V
		FYCO1	V	V					V	V
		CCR9	V					V	V	V
		CXCR6	V					V	V	
		XCR1	V					V		
6p21.33	rs143334143	CCHCR1		V			V	V	V	
		TCF19		V						V
12q23.2	rs10860891	IGF1					V	V	V	
19p13.3	rs2277732	DPP9	V	V	V	V	V	V	V	V
		HDGFL2				V		V		V
7p11.2	rs622568	VSTM2A		V	V			V	V	
21q22.11	rs13050728	IFNAR2	V	V	V	V		V	V	V
		IL10RB	V		V	V		V	V	
		IFNAR1				V		V	V	V
6p21.1	rs1853837	FOXP4	V	V		V			V	
19p13.2	rs11085727	TYK2		V	V	V	V	V		
		FDX2			V			V		
		ICAM4				V		V		
12q24.13	rs4766664	OAS1		V			V		V	
		OAS2			V				V	
		OAS3	V	V	V	V	V	V	V	
9q34.13	rs8176719	ABO	V							V
		OBP2B		V						V
9q21.31	rs117797075	TLE1					V			V

**Table 2:** Approved druggable drug-gene interactions for COVID-19 drug repurposing

Target	Generic Name (DrugBank ID)	Ligand class	Mechanism of action	Action	COVID-19 related pathway
IFNAR1, IFNAR2	Peginterferon alfa-2b (DB00022)	Anti-HBV, HCV	binds to type I interferon receptors (IFNAR1)	agonist	SARS-CoV-2 have evolved mechanisms to evade the antiviral function of type I interferon (IFN-1)
IFNAR1, IFNAR2	Interferon alfa-2b (DB00105)	Anti-HBV, HCV	Interferon-beta binds to type I interferon receptors (IFNAR1 and IFNAR2c) which activate two Jak (Janus kinase) tyrosine kinases (Jak1 and Tyk2)	agonist	SARS-CoV-2 have evolved mechanisms to evade the antiviral function of type I interferon (IFN-1)
IFNAR1, IFNAR2	Interferon beta-1b (DB00068)		Interferon-beta binds to type I interferon receptors (IFNAR1 and IFNAR2c) which activate two Jak (Janus kinase) tyrosine kinases (Jak1 and Tyk2)	agonist	SARS-CoV-2 have evolved mechanisms to evade the antiviral function of type I interferon (IFN-1)
TYK2	Ruxolitinib (DB08877)	Immunosuppressants	Janus kinase (JAK) inhibitor	inhibitor	host-driven inflammatory lung injury, which is a crucial mechanism of late, life-threatening COVID-19

**Table 3:** Highly druggable drug-gene interactions for COVID-19 drug repurposing from several supported evidences

Target	Phenotypes of COVID-19 from GWAS	Ligand name (DrugBank ID)	Ligand class	Mechanism of action	Binding affinity (kcal/mol)	COVID-19 relevant reference
IL10RB	Critical illness, Hospitalized	Dactinomycin (DB00970)	antineoplastics	Inhibition of viral cellular transcription	−13.5	^32^
CXCR6	Critical illness, Hospitalized	Rolitetracycline (DB01301)	Antibacterials	bind to bacterial 30S ribosomal subunit	−16.1	^39^
CCR9	Critical illness, Hospitalized	Irinotecan (DB00762)	Antineoplastics	inhibit DNA topoisomerase 1	−17.1	^40^
XCR1	Critical illness	Vinblastine (DB00570)	Antineoplastics	inhibit tubulin mitotic functioning	−16.3	^35^
OAS1	Hospitalized	ORITAVANCIN (DB04911)	antibacterials	Inhibition of cathepsin L and cathepsin B in host cells (late endosomal pathway)	−16.6	^33^

## Data Availability

All COVID-19 GWAS meta-analyses data utilized in this analysis can be accessed at the COVID-19 Host Genetics Initiative: https://www.covid19hg.org/ All docking results are stored in the DockCoV2 database: https://dockcov2.org/drugs/.
